# Optimized Home Rehabilitation Technology Reduces Upper Extremity Impairment Compared to a Conventional Home Exercise Program: A Randomized, Controlled, Single-Blind Trial in Subacute Stroke

**DOI:** 10.1177/15459683221146995

**Published:** 2023-01-12

**Authors:** Veronica A. Swanson, Christopher Johnson, Daniel K. Zondervan, Nicole Bayus, Phylicia McCoy, Yat Fung Joshua Ng, Jenna Schindele, BS, David J. Reinkensmeyer, Susan Shaw

**Affiliations:** 1 Department of Mechanical and Aerospace Engineering, Henry Samueli School of Engineering, University of California, Irvine, Irvine, CA, USA; 2 Department of Biomedical Engineering, Henry Samueli School of Engineering, University of California, Irvine, Irvine, CA, USA; 3 Flint Rehabilitation Devices, LLC, Irvine, CA, USA; 4 Rancho Research Institute, Rancho Los Amigos National Rehabilitation Hospital, Downey, USA; 5 Arthur J. Bond Department of Mechanical Engineering, Alabama A&M University, Huntsville, AL, USA; 6 School of Social Sciences, University of California, Irvine, Irvine, CA, USA; 7 Mathematics and Statistics, University of California, Los Angeles, Los Angeles, CA, USA; 8 Department of Anatomy and Neurobiology, UC Irvine School of Medicine, University of California, Irvine, Irvine, CA, USA; 9 Department of Neurology, Rancho Los Amigos National Rehabilitation Center, Downey, CA, USA

**Keywords:** stroke, rehabilitation, exercise therapy, home exercise program, FitMi, mRehab, sensors

## Abstract

**Background:**

Upper extremity (UE) stroke rehabilitation requires patients to perform exercises at home, yet patients show limited benefit from paper-based home exercise programs.

**Objective:**

To compare the effectiveness of 2 home exercise programs for reducing UE impairment: a paper-based approach and a sensorized exercise system that incorporates recommended design features for home rehabilitation technology.

**Methods:**

In this single-blind, randomized controlled trial, 27 participants in the subacute phase of stroke were assigned to the sensorized exercise (n = 14) or conventional therapy group (n = 13), though 2 participants in the conventional therapy group were lost to follow-up. Participants were instructed to perform self-guided movement training at home for at least 3 hours/week for 3 consecutive weeks. The sensorized exercise group used FitMi, a computer game with 2 puck-like sensors that encourages movement intensity and auto-progresses users through 40 exercises. The conventional group used a paper book of exercises. The primary outcome measure was the change in Upper Extremity Fugl–Meyer (UEFM) score from baseline to follow-up. Secondary measures included the Modified Ashworth Scale for spasticity (MAS) and the Visual Analog Pain (VAP) scale.

**Results:**

Participants who used FitMi improved by an average of 8.0 ± 4.6 points on the UEFM scale compared to 3.0 ± 6.1 points for the conventional participants, a significant difference (*t*-test, *P* = .029). FitMi participants exhibited no significant changes in UE MAS or VAP scores.

**Conclusions:**

A sensor-based exercise system incorporating a suite of recommended design features significantly and safely reduced UE impairment compared to a paper-based, home exercise program.

**Trial Registration:**

ClinicalTrials.gov Identifier: NCT03503617

## Introduction

Stroke is a leading cause of chronic disability in the United States.^1-3^ Intensive upper extremity (UE) rehabilitation can reduce long-term impairment after stroke^4-9^ and is more effective when delivered in the subacute phase following stroke.^10^ Though exact dose-response relationships are unknown, recovery also appears to depend on the volume of movement practice.^11-15^ One-on-one supervised therapy sessions are likely insufficient to achieve the required dose.^16,17^ Home exercise programs are prescribed to increase movement training dose, but the current standard of care—following printed sheets of exercises—is associated with poor compliance, poorer outcomes, and high dropout rates.^18-23^

Recognizing the need for sustainably increasing the amount of movement practice that individuals undertake, there has been a surge in the development of technologies for enabling individuals to practice on their own at home.^20,24-32^ Home-based technologies for stroke rehabilitation include sensors, games, telerehabilitation, robotic devices, virtual reality, apps, and tablets.^33^ As technologies have been developed and tested, a set of recommended design features has emerged (Table 1). However, the net effect of optimizing home rehabilitation technology by implementing these features is still unclear.

**Table 1.** Summary of Recommended Design Features for Home Rehabilitation Technology.

Domain	Recommendation
Hardware design	System is small, lightweight, easy to store, portable^33,34-38^
	Hardware is adjustable for different body sizes and different grip types or movements^34^
	Sensors are reliable and validated sensor accuracy^33,34-36^
	System can be interfaced with the user’s existing TV, computer, or mobile device^39^
	System is robust and not easily damaged^37,38^
	Hardware promotes quality movements and helps prevent compensatory motions^37,39^
Software interface design	Software is easy to navigate, with clear and simple operating instructions^34,35,39^
	Software provides a tutorial or introduction to use^34^
	Software contains clear text displayed in a large font size^35^
Operation	System requires simple installation, setup, shut down, and charging procedures^33,34,37^
	System is simple enough to be used with minimal external support^34,37,38,40^
Therapeutic activity design	Activities are physically challenging but also achievable^34,37,39,41^
	Activities incorporate games or gamification to enhance motivation^33^
	Activities are tailored to personal goals, needs, and interests^33,42^
	System includes a variety of activities that accommodate different ability levels^33,34,37,39^
	Difficulty and duration adapt as user improves, both over time and in the moment^33,40^
	Movements practiced relate to functional movements or activities of daily living (ADL)^34,39^
Performance feedback	Feedback is multi-modal (eg, numerical, graphical, and auditory)^34,39,40^
	Feedback on performance is provided during the activity^35,39^
	Summary feedback is presented immediately after the activity is complete^34,35,37,39^
	A history of the user’s performance over time is available^33,34,35,39^
	System enables goal tracking^35^
	System encourages periods of rest when applicable^37^
	System provides positive feedback with a partial reinforcement schedule^37^
	A healthcare professional can monitor the user remotely and provide feedback^34,39,40^
Support	Technical support is available, especially at start of use^33^
	Support is offered using multiple communication methods (eg, text, voice, and video)^39^
	Support is available in different languages^39^
	System enables healthcare providers to communicate with user^39^
Safety	An emergency stop button and/or warning messages are provided when appropriate^34^
	Hardware design avoids sharp edges, possible finger traps, and protects the users’ skin^34^
Cost	System is relatively low cost^37,38,41^
User’s social context	System is attractive and acceptable to the user and their family members^33^
	System does not create additional burden on family members or caregivers^37^
	System can be used cooperatively with a family member or friend^39^

FitMi was designed to incorporate most of these features, except for the 4 that are italicized, which can be grouped into 2 categories: ensuring that high-quality movements are practiced and facilitating collaboration with a therapist or caregiver. We generated this table based on a systematic review of recommendations,^34^ but also incorporated suggestions from other studies of home rehabilitation technology that were not included in that review, as indicated by the referencing.

**Figure 1.**
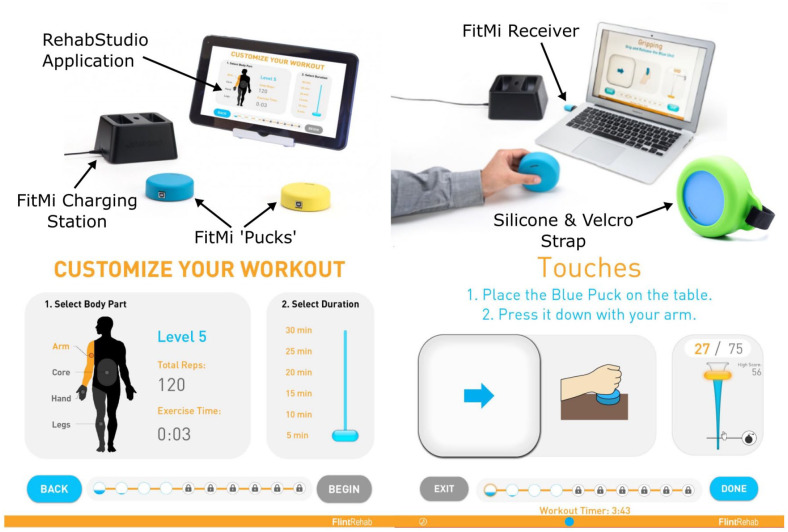
FitMi (produced by Flint Rehab, LLC) consists of 2 force and motion sensing pucks and a companion “mixed-reality gym” software application. Top row: FitMi hardware. Bottom row: FitMi software. Note, FitMi can be used with an individual’s existing computing hardware (Top Right) or with a custom 10″ touchscreen tablet in a kiosk mode that only requires users to turn the tablet on and touch an icon to access the application (Top Left).

FitMi (Figure 1) is a commercial home rehabilitation technology designed to put into practice many of these features (see Table 1). This randomized controlled trial aimed to evaluate the effectiveness of FitMi in reducing UE impairment compared to conventional paper-based home exercises in the subacute phase following a stroke. We hypothesized that the participants in the FitMi group would improve their Upper Extremity Fugl–Meyer (UEFM) score significantly more than the conventional therapy group, as assessed at the follow-up assessment.

## Methods

### Device Design

The FitMi hardware consists of 2 wireless input devices (called pucks), a USB receiver, a docking station for one-handed charging, and a silicone strap for users who have difficulty grasping the pucks (see Figure 1). Each puck contains an accelerometer, gyroscope, magnetometer, load cell, onboard LED, and vibration motor. The top half of the puck is coupled to the bottom half through the load cell, allowing the device to detect either pressing forces or grip forces. Data from each puck’s sensor array is wirelessly transmitted to the USB receiver, which can be plugged into any computer and used without configuring the devices. Using this data and custom software algorithms, FitMi detects the completion of 40 different exercises for the hands, arms, trunk, and legs that were designed with the input of experienced stroke therapists (Supplemental Text 1). For each exercise, the FitMi software presents users with a repetition goal for a bout of exercise, progress toward that goal, and real-time feedback each time a repetition is completed. Before exercising, users are shown written instructions and images of the starting and ending position for each repetition of the given exercise. They can also watch a video of an experienced therapist demonstrating the exercise and providing tips to prevent compensatory movement patterns. Once users begin an exercise, the screen indicates the exercise position they need to move toward (Figure 1), and the system provides a game-like environment with music that encourages movement intensity. Users are provided with audio, visual, and haptic feedback as they repetitively move between the starting and ending positions for each exercise. The height of an exercise intensity bar indicates their exercise rate, and if the rate slows too much, the bar hits a “bomb,” and the exercise session ends. After an exercise is completed, the software displays the user’s performance history over time, both within an exercise session and across days of use. To optimize the system’s challenge level, the software progressively unlocks new exercises and adapts the goal number of repetitions for each exercise based on the user’s past performance. At any time, users can access an interactive tutorial on how to use the software. The FitMi software can run on a personal computer or a custom 10″ touchscreen tablet in a kiosk mode (ie, the tablet runs no other software besides the FitMi software).

### Trial Design

This study was a single-site, single-blind randomized controlled trial comparing home-based therapy with FitMi to conventional therapy for individuals in the subacute phase of stroke. The study was performed at Rancho Los Amigos National Rehabilitation Center in Downey, CA. Participants were invited for an initial assessment to confirm they met the inclusion criteria and to establish baseline measures. Participants provided informed written consent. Qualifying participants were randomly assigned to either the FitMi group or the conventional group. Participants in both groups were instructed to perform self-guided therapy for at least 3 hours/week for 3 consecutive weeks. All participants received weekly phone calls from a supervising therapist. After the 3-week exercise period, participants returned for an end-of-therapy assessment and to return study materials. Participants returned 1 month later for a follow-up assessment. The trial was pre-registered on ClinicalTrials.gov (NCT03503617) and approved by the Rancho Research Institute, Inc. Institutional Review Board at Rancho Los Amigos National Rehabilitation Center (IRB #263).

### Participants

Inclusion criteria were: experienced one or more strokes between 2 weeks and 4 months prior; baseline UEFM Score >5 and ≤55 out of 66; absence of moderate to severe pain defined as a score of 4 or lower on the 10-point visual-analog pain scale; ability to understand the instructions to operate FitMi; and aged 18 to 85 years old, to limit potential confounds due to naturally diminished physical mobility and cognitive function associated with older age.^43^ Exclusion criteria were: concurrent severe medical problems that precluded the individual from participating in routine rehabilitation; visual deficits defined as a score >1 on question 3 of the NIH Stroke Scale (NIHSS); severe cognitive deficits or apraxia defined as a score >0 on questions 1a and 1c of the NIHSS; severe neglect defined as a score >1 on question 11 of the NIHSS; severe aphasia defined as a score >1 on question 9 of the NIHSS; and enrollment in other therapy studies. Recruitment aimed to balance the age, ethnicity, and gender of the study participants to be representative of Los Angeles County in California, USA. All participants provided informed consent.

Using an estimated Cohen’s *d*^44^ effect size of 1.05 based on long-term follow-up data from a previous arm training study during subacute stroke,^45^ power analysis established that 21 participants in each group would provide a 90% chance of detecting a significant difference between FitMi and conventional therapy at the .05 significance level (two-tailed *t*-test). To account for 20% dropout, the target sample size was n = 25 participants in each group.

Adaptive randomization was used to ensure matched levels of impairment between the FitMi and conventional therapy groups. Specifically, subjects were stratified by their UEFM Score into 3 levels (ie, 5-22, 23-39, 40-55) and then randomized by alternating block allocation.^46^

### Intervention

Participants randomized to the FitMi group were given a FitMi system with a custom 10″ touchscreen tablet. They received 30 minutes of training on how to set up and use the FitMi system. They were instructed to spend most of their time performing UE exercises, but access to the trunk and leg exercises in the FitMi software was not disabled. Participants randomized to the conventional therapy group were given a booklet of paper exercises that were selected from the same library of 40 exercises available in the FitMi software. The booklet was placed in a sensorized folder which included an accelerometer to detect movement events and a magnetometer and magnet on opposite sleeves to detect when the folder was opened or closed. These events were recorded to a memory card by an embedded microcontroller.

For both groups, a supervising rehabilitation therapist selected the exercises for each participant based on their specific impairments. All participants received 30 minutes of training from the therapist on how to perform the selected exercises correctly. After the 3-week exercise period, participants returned for an end-of-therapy assessment. At this assessment, participants returned the FitMi system or the sensorized booklet of exercises for data collection. Participants returned 1 month later for a follow-up assessment.

### Outcomes

The primary outcome measure was the change in UEFM score^47^ from baseline assessment to follow-up. UEFM was assessed at baseline, end-of-therapy, and follow-up. Secondary measures included the Box and Blocks Test,^48^ the 10 Meter Walk Test,^49^ the Modified Ashworth Spasticity (MAS) scale^50^ for the elbow, wrist, and fingers, and the Visual Analog Pain (VAP) scale for the UE, all of which were assessed at baseline, end-of-therapy, and follow-up. Motor Activity Log (MAL) was measured at end-of-therapy and follow-up to assess self-reported quantity and quality of movement.^51^ The European Quality of Life five dimensions, three levels (EQ-5D-3L) and its companion Visual Analog Scale (EQ-VAS) were measured at end-of-therapy and at follow-up to assess overall perceived health state,^52,53^ and the Intrinsic Motivation Inventory (IMI)^54^ categories of Interest/Enjoyment, Value/Usefulness, and Effort/Importance were measured at end-of-therapy to assess participants’ perceived motivation. These measures are widely used in stroke rehabilitation research and have good sensitivity and reliability. All assessments were performed by a blinded, trained evaluator.

To assess adherence, the FitMi software recorded the date, time, and number of repetitions completed for each exercise, and the sensorized folder used in the conventional therapy measured the times at which the participants opened the booklet.

### Statistical Methods

Statistical analyses were performed using Matlab R2020 software. For measures taken at baseline and follow-up, the change from baseline to follow-up was calculated. Then the changes were compared between groups using an unpaired two-tailed *t*-test. This was the analysis specified for the primary outcome in the statistical analysis protocol established before the project started. For the UEFM, we also assessed within-group changes between timepoints using paired *t*-tests. We corrected for multiple comparisons for secondary outcomes using a Holm-Bonferroni correction. Cohen’s *d*, using pooled standard deviation, was used to assess the effect size of the difference in changes between groups. As a post-hoc, supplemental analysis, mixed model ANOVAs were used to analyze outcomes taken at all 3 time points, using the mixed procedure in SPSS 28.0, to further account for variance over time. If a significant time and group interaction was found, pairwise comparisons were then used to find differences within or between groups at any time point by using Sidak adjustments to correct for multiple comparisons.

MAS scores were grouped by flexion or extension items and summed to obtain lumped MAS extension and flexion values. We quantified items marked with a “+,” with an additional 0.5 points for calculations. EQ-5D-L3 was analyzed following.^52,53^ Responses to questions in IMI categories were averaged within the category for each participant and compared across groups using Wilcoxon rank sum tests.

**Figure 2.**
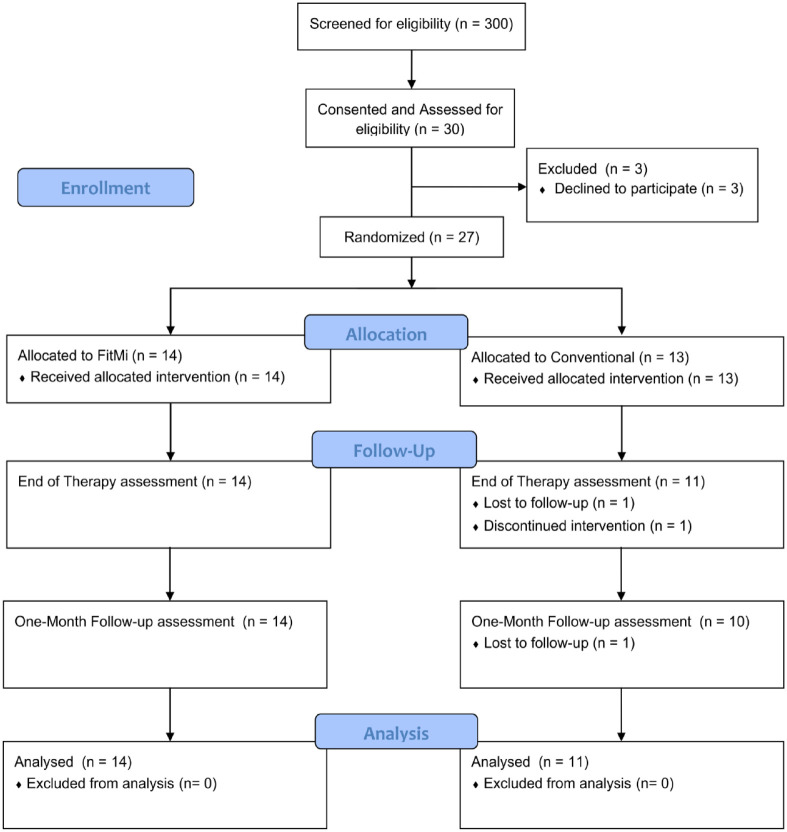
Participant flow diagram. Subjects who did not return for an end-of-therapy assessment were not considered for analysis. Missing data was imputed as described in the statistical methods to maintain group sizes across all analyses.

Several participants dropped out of the study (see Figure 2). Subjects who did not return for an end-of-therapy assessment were not considered for analysis. Missing data was imputed to keep the same number of participants across timepoints for analysis using the MissForest random forest imputation algorithm.^55^

To assess the ability of FitMi to motivate an appropriately high dose of home therapy, we performed a post-hoc exploratory analysis comparing the total number of repetitions that FitMi participants completed to a theoretical target dose of 2700 repetitions. A dose of 2700 repetitions of UE exercise corresponds to 300 repetitions/hour (5 reps/minute) over 9 hours of exercise, an intensity and duration sufficient to provoke a forelimb rehabilitative effect in a rodent model of stroke.^56^

### Interim Analysis

Due to the unexpected additional risks to participating in this study due to the COVID-19 pandemic, an unplanned interim futility/efficacy analysis of the primary outcome measure was conducted after recruitment was halted in March 2020. Group labels were removed, and the analysis was reviewed by an independent investigator. For the futility analysis, a conditional power of 20% was selected. For the efficacy analysis, a *P*-value of .033 was selected using the Lan-DeMets alpha spending function for the Pocock boundary (n = 27 out of a planned 50 at interim analysis).^57^

## Results

### Recruitment and Participant Flow

Participants were recruited from November 20, 2018, until March 12, 2020, when the study was halted due to the COVID-19 pandemic. In the interim analysis, a significant difference in the primary outcome measure was observed between groups (two-tailed *t*-test, *P* < .033). Thus, recruitment was halted early based on detected efficacy at 27 out of a planned 50 participants.

Participant enrollment and allocation details are shown following CONSORT guidelines in Figure 2. Out of 300 individuals screened for eligibility, 27 were randomized (Table 2). Two participants from the conventional therapy group did not complete the end-of-therapy or follow-up assessment due to a second stroke for one and COVID-19 restrictions for the other. An additional conventional therapy participant could not return for the follow-up due to COVID-19 restrictions.

**Table 2.** Demographics of Recruited Participants at Baseline Aggregated by Group.

	Control	FitMi
Number of participants	13	14
Age (y)	52 ± 8.7	50.3 ± 10.9
Sex (M/F)	9/4	14/0
Ethnicity (H/N)	10 H, 3 N	8 H, 6 N
Stroke type (I, H, B)	9 I, 3 H, 1 B	11 I, 3 H
Impaired side (L/R)	8 L, 5 R	10 L, 4 R
Number dominant side impaired	5	4
Weeks post stroke	10.1 ± 5.1, [4.6, 17.6]	9.7 ± 4.5, [4.3, 17.9]

Abbreviations: H, Hispanic/Latino; N, not Hispanic/Latino; I, ischemic; H, hemorrhagic; B, both ischemic and hemorrhagic.

Where applicable, values are reported as Mean ± SD, [minimum, maximum]. Weeks Post Stroke indicates the number of weeks between the participant’s stroke and the date of their baseline evaluation.

### Efficacy

All measures recorded at baseline, end-of-therapy, and follow-up assessments are reported in Table 3. All measures not recorded at baseline but recorded at end-of-therapy and follow-up are reported at the bottom of Table 3. UEFM scores at baseline ranged from 12 to 53 for the FitMi group and 9 to 50 for the conventional therapy group, indicating enrollment across a broad range of motor impairments (Supplemental Figure 1). There was no significant difference in the UEFM score, or any other outcomes, between groups at baseline (*P* > .3).

**Table 3.** Results for Outcome Measures for FitMi and Conventional Therapy Groups.

	BL	EOT	FU	Δ from BL to FU	*p*-Value between-group comparisons of Δ	Effect size
UEFM						
FitMi therapy	36.7 ± 15.4	43.2 ± 16.3	44.7 ± 16.2	8.0 ± 4.6	.029^a^	0.925
Conventional therapy	35.18 ± 14.5	36.64 ± 14.8	38.18 ± 16.2	3.0 ± 6.1		
Box and blocks						
FitMi therapy	25.4 ± 17.6	28.9 ± 17.6	30.2 ± 19.7	4.8 ± 6.3	.701	0.156
Conventional therapy	23.5 ± 14.8	24.5 ± 15.8	27.2 ± 16.1	3.7 ± 7.2		
10 meter walk test (m/s)						
FitMi therapy	0.98 ± 0.37	1.02 ± 0.40	1.06 ± 0.41	0.08±0.020	.857	0.071
Conventional therapy	0.86 ± 0.28	0.87 ± 0.40	0.92 ± 0.48	0.06 ± 0.33		
MAS (extension)						
FitMi therapy	0.57 ± 0.87	0.50 ± 0.71	0.46 ± 0.69	−0.11 ± 0.59	.944	0.029
Conventional therapy	0.55 ± 1.04	0.18 ± 0.40	0.45 ± 0.82	−0.09 ± 0.54		
MAS (flexion)						
FitMi therapy	2.8 ± 1.9	2.4 ± 2.2	2.5 ± 2.0	−0.29 ± 1.59	.828	0.091
Conventional therapy	2.4 ± 2.1	1.6 ± 1.5	2.0 ± 2.2	−0.41 ± 1.10		
VAP						
FitMi therapy	1.3 ± 1.7	2.1 ± 2.4	2.6 ± 2.1	1.4 ± 2.3	.176	0.555
Conventional therapy	1.5 ± 1.5	4.0 ± 3.4	4.3 ± 2.8	2.8 ± 2.9		
		EOT	FU			
MAL (AS)						
FitMi therapy		2.81 ± 0.74	3.11 ± 1.14			
Conventional therapy		2.52 ± 1.74	2.41 ± 1.82			
MAL (HW)						
FitMi therapy		2.78 ± 1.01	3.01 ± 1.26			
Conventional therapy		2.22 ± 1.58	2.26 ± 1.71			
EQ-5D-3L						
FitMi therapy		0.77 ± 0.10	0.74 ± 0.06			
Conventional therapy		0.84 ± 0.09	0.82 ± 0.11			
EQ-VAS						
FitMi therapy		63.93 ± 17.24	74.2 ± 15.6			
Conventional therapy		71.36 ± 14.16	70.27 ± 10.90			

Abbreviations: BL, baseline; EOT, end-of-therapy; FU, follow-up; UEFM, Upper Extremity Fugl–Meyer; MAS, Modified Ashworth scale for spasticity; VAP, visual analog pain; MAL, motor activity log; EQ-5D-3L, European Quality of Life five dimensions, three levels; EQ-VAS, EuroQol Visual Analog Scale.

For measures with a baseline assessment, the change from baseline to follow-up was calculated. For each measure, the change was compared between groups using unpaired, two-tailed *t*-tests. *T*-tests for secondary outcomes were corrected for multiple comparisons using a Holm-Bonferroni correction. The difference between groups was quantified using Cohen’s d effect size. The absolute value of the effect size is shown here.

a Indicates a significant difference using the corrected α-value for that assessment.

**Figure 3.**
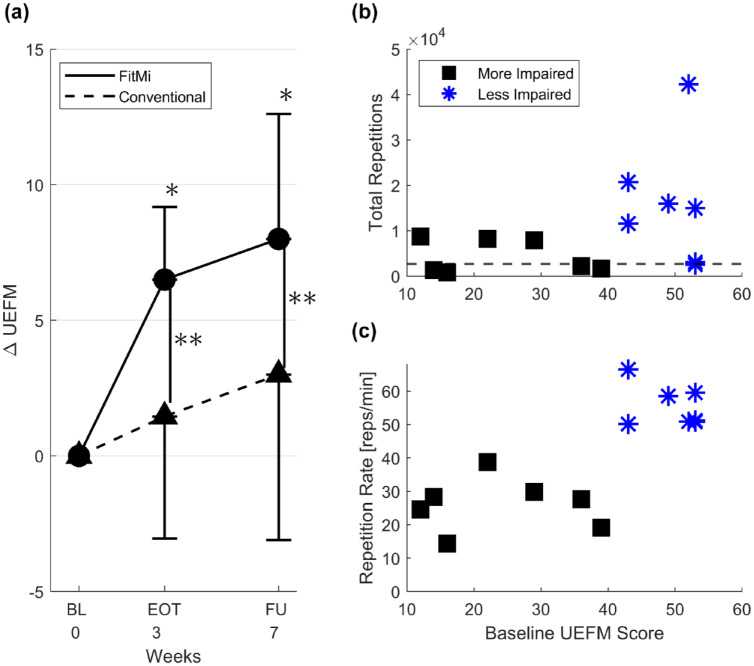
(A) Average change in Upper Extremity Fugl–Meyer (UEFM) score for FitMi group and conventional therapy group at each assessment. The error bars represent 1 SD. (BL = Baseline) FitMi participants improved significantly more than the conventional therapy group. *Indicates a significant within-group difference between UEFM scores at each time point compared to baseline (*p* < .01). **Indicates a significant difference between the change in UEFM between groups at each time point (*p* < .033). (B) FitMi participants’ total number of repetitions plotted as a function of their baseline UEFM score. The horizontal dashed line indicates a theoretical target dose of 2700 repetitions. Note, there are 2 overlapping participants at baseline UEFM score 53, one who exceeded the threshold and one who did not. (C) FitMi participants’ average repetition rate, in repetitions per minute, plotted as a function of their baseline UEFM score. Abbreviations: EOT, end-of-therapy; FU, follow-up.

For the primary outcome measure, the average change in UEFM from baseline to follow-up for participants in the FitMi group (n = 14) was 8.0 ± 4.6 compared to an average change of 3.0 ± 6.1 for participants in the conventional therapy group (n = 11), a significant difference with a large effect size (*P* = .029, *d* = 0.925; Table 3). A significant within-group increase in UEFM score was found for the FitMi group when comparing baseline to end-of-therapy and follow-up (*P* < .001 for both intervals), but not for the conventional therapy group at either assessment (Figure 3).

The 2 groups’ scores did not change significantly differently for any of the secondary outcomes.

Mixed model ANOVA analysis for UFEM scores found a significant time effect (*F*(1.771, 40.725) = 21.119, *P* < .001, ηp^2^ = .479), and a significant time × group interaction (*F*(1.771, 40.725) = 5.506, *P* = .010, ηp^2^ = .193), but not a significant group effect. Pairwise comparisons show the FitMi group’s UEFM scores increased significantly between baseline and end-of-therapy (*P* < .001), and between baseline and follow-up (*P* < .001) but did not significantly change between end-of-therapy and follow-up. Significant time effects were found for the Box and Blocks assessment (*F*(1.718, 39.511) = 7.376, *P* < .002, ηp^2^ = .243) and the VAP (*F*(2, 46) = 9.175, *P* < .001, ηp^2^ = .285), but neither of these assessments showed significant time × group interactions or group effects. No significant effects were found for the 10 m walk test, MAS Extension, or MAS Flexion. Sphericity was violated for the UEFM and Box and Blocks scores, so the Huynh-Feldt corrected results are reported for these assessments.

### Safety and Motivation

No significant harms related to the study were reported or observed over the course of the study. For participants in the FitMi therapy group, no significant change was found between baseline and end-of-therapy for MAS or VAP scores (paired *t*-test *P* > .05). No significant difference was found between the FitMi therapy and conventional therapy participants in their responses to IMI questions related to Interest/Enjoyment (FitMi 5.1 ± 1.1, Conventional 5.4 ± 1.1), Value/Usefulness (FitMi 6.5 ± 0.7, Conventional 6.3 ± 0.6), and Effort/Importance (FitMi 6.4 ± 1.5, Conventional 6.7 ± 0.6). All items are rated on a scale from 0 to 7.

Participants interacted with the FitMi software for a median of 47% of the 21 days of the intervention period (range = 23%-100%). FitMi participants interacted with the system for 5.4 ± 4.1 hours. Only 2 participants completed or exceeded the recommended 9 hours of interaction time. Due to technical issues with battery life, only 4 out of 13 sensorized folders provided to participants in the conventional therapy group were returned with recoverable data. These 4 participants interacted with their folders for 41%, 73%, 45%, and 100% of the 21 days of the intervention.

Of the 14 participants in the FitMi group, 9 out of 14 (64%) completed the theoretical target dose of at least 2700 repetitions (as defined in the Methods) over 3 weeks of exercise, with 7 participants completing more than 3 times this amount (Figure 3B). Across the 4 exercise categories available in the FitMi software (hand, arm, trunk, and leg exercises), the participants completed 4051 ± 4986 [130, 18 617], 2744 ± 3076 [186, 11 262], 1550 ± 1439 [176, 5469], 1813 ± 2029 [0, 6908] repetitions, respectively (reported as mean ± standard deviation [minimum, maximum]).

We tested whether less impaired participants achieved more repetitions with FitMi. To do this, we ranked participants by baseline UEFM score and then split them into evenly sized groups (n = 7) thus creating lower and higher UEFM groups defined by a UEFM cutoff of 40. Comparing total repetitions between groups (4427 ± 3648 [832, 8694] vs 15 890 ± 13 412 [2565, 42 256], respectively) revealed a significantly greater amount of exercise in the higher UEFM group (unpaired *t*-test, *P* = .0497). In terms of exercise rate, the lower UEFM group exercised more slowly (26 ± 8 reps/minute) than the higher UEFM group (55 ± 6 reps/minute) (unpaired *t*-test, *P* < .001) (Figure 3C). The change in UEFM score from baseline to end-of-therapy was moderately correlated with the total number of arm and hand repetitions each participant achieved (*P* = .005, *r*^2^ = .52, Supplemental Figure 2); the correlation was not significant at follow-up (*P* = .11, *r*^2^ = .22, Supplemental Figure 3). The participant with the highest number of repetitions was omitted as an outlier for these correlation analyses.

## Discussion

We compared the effectiveness of a sensorized exercise system, FitMi, with a conventional exercise program specified using a paper booklet for at-home movement training in subacute stroke. Participants who exercised with FitMi improved significantly more on the primary outcome, the change in the UEFM scale from baseline to follow-up, compared to the participants in the conventional therapy group without increasing UE spasticity or pain. We first discuss the significance of these results, followed by limitations and directions for future research.

### Toward Optimizing Home Rehabilitation Technology

As reviewed in Table 1, FitMi was designed in a way consistent with previous research that recommended desirable features for home rehabilitation technology. The current study provides evidence that these features, when bundled together, make home movement training more effective at reducing UE impairment compared to the conventional, paper-based prescription of exercise in subacute stroke. Although the core mechanisms remain unclear, we speculate that this result relates to 2 causes. We hypothesize that the FitMi participants achieved more movement repetitions than the conventional therapy group because FitMi participants likely exercised at a higher intensity. Specifically, the FitMi technology encouraged rapid repetition of exercise and adaptively progressed the goal number of repetitions for each exercise based on each user’s past performance—something that paper exercises cannot do. Indeed, the average exercise rates were quite high compared to what might be expected with paper-based exercise, being 26 reps/minute for the lower UEFM group and 55 reps/minute for the higher UEFM group. Interestingly, the IMI scores did not indicate that subjective self-report of motivation was significantly higher for the FitMi group. This may be because both groups had high motivation to achieve recovery at this early stage regardless of intervention type. Future studies could focus on understanding the motivation to recover versus the motivation to exercise at a high intensity.

An important question is whether the system was usable by more severely impaired individuals, as there are fewer options available for such persons for continuing movement practice. The lower half of participants with more severe impairments (UEFM <40) still achieved on average 4427 repetitions, an amount that exceeded the theoretical target dose of 2700 repetitions. Notably, most participants were able to exceed the 2700-repetition target in a shorter amount of time than was prescribed, because they achieved an average rate of exercise of 41 ± 17 reps/minutes, which was greater than the 5 reps/minute we estimated a priori. This indicates that FitMi was accessible and motivating for individuals with a range of impairment levels, which is a key requirement for optimizing home rehabilitation technology as it allows a single solution to be used across a broad population.

As shown in Table 1, FitMi does not currently incorporate design features to (1) ensure that only high-quality movements can be practiced, or (2) facilitate collaboration with a therapist or caregiver. A key reason these features were previously recommended was to ensure that patients do not practice unsafe or compensatory movement patterns during unsupervised at-home therapy. However, in the present study, we found no significant increase in spasticity or pain in the FitMi group. Further, the observed reduction in UE impairment in the FitMi group cannot be explained by the learning of compensatory movements, since compensatory movements are discounted in the UEFM scoring process. Thus, foregoing these features in FitMi’s design did not appear to reduce safety or encourage abnormal movement execution in the present study. Nonetheless, incorporating these features into FitMi’s design might improve future results.

While home training with FitMi led to a significantly greater reduction in UE impairment than paper-based exercise, an important question is whether the amount of improvement was clinically significant. The Minimal Clinically Important Difference (MCID) for the UEFM has been reported to be 4 points for subacute patients,^58^ although another study with younger patients (average age 52) closer to the mean age of the participants in our study estimated it to be 9 points in the first few weeks (4-24 weeks) after stroke.^59^ The MCID was reported to be ~5 points in the chronic stage of stroke for older patients.^60^ Six out of 14 (43%) of participants in the FitMi group achieved a 9-point change in UEFM (with 2 additional participants achieving 8-point changes) compared to 2 out of 10 (20%) of participants in the conventional therapy group (with none achieving 8-point changes). Thus, exercise with FitMi appears to have a clinically meaningful impact for more individuals than paper-based exercise.

### Limitations and Future Directions

No female participants were recruited into the FitMi therapy group, which limits the generalizability of the reported results. A smaller percentage of FitMi participants were impaired on their dominant side than in the conventional group (29% vs 38%). Alternating allocation has been shown to be prone to selection bias^61^ and does not allow for naïve allocation. The randomization procedure used was peer-reviewed and approved before the study began, and analysis of group characteristics at baseline did not reveal any statistically significant differences between group characteristics. However, future protocols could be improved by using different randomization methods. While the FitMi and conventional therapy group’s clinical assessment scores were matched at baseline, we did not evaluate possible differences in their potential for recovery using biomarkers such as motor-evoked potentials.^62,63^ Recruitment was also stopped early due to the COVID-19 pandemic, reducing statistical power. Several participants who were recruited dropped out of the study (most due to COVID-19 restrictions, and all from the conventional therapy group) and required multiple imputation for analysis. Finally, while the number of days participants in the FitMi group exercised had a similar distribution to the 4 participants in the conventional therapy group for whom we collected data from their sensorized folders, we did not quantify the number of exercise repetitions participants in the conventional therapy group achieved. This limits our ability to determine if the observed benefits of FitMi are simply due to a higher number of movements performed or a specific benefit of the FitMi device.

Future research could study how exercise technologies such as FitMi can best be integrated into routine clinical practice. Providing stroke survivors with FitMi in any waiting period between the end of their inpatient treatment and the start of their outpatient treatment, or after they have used all the outpatient therapy visits allotted by their health insurance, could improve outcomes. We recently studied the use of the FitMi sensors in conjunction with an activity-management app to assist in home rehabilitation.^64^ Therapists reported that remote monitoring and the use of a physical movement sensor were motivating to their patients and increased adherence. We also recently studied the long-term, self-determined exercise patterns of a large number of individuals (N = 2581) who engaged in home rehabilitation with FitMi. We found that an optimized challenge level and regular initiation of exercise sessions predicted the achievement of a greater amount of overall rehabilitation exercise.^65^ Going forward, the fine-grained data collection facilitated by an accessible, commercially available, sensorized home exercise system such as FitMi opens interesting avenues of analysis to investigate the effects of the amount and type of exercise on rehabilitation outcomes in the real world.

## Supplemental Material

sj-doc-5-nnr-10.1177_15459683221146995 – Supplemental material for Optimized Home Rehabilitation Technology Reduces Upper Extremity Impairment Compared to a Conventional Home Exercise Program: A Randomized, Controlled, Single-Blind Trial in Subacute Stroke Click here for additional data file. Supplemental material, sj-doc-5-nnr-10.1177_15459683221146995 for Optimized Home Rehabilitation Technology Reduces Upper Extremity Impairment Compared to a Conventional Home Exercise Program: A Randomized, Controlled, Single-Blind Trial in Subacute Stroke by Veronica A. Swanson, Christopher Johnson, Daniel K. Zondervan, Nicole Bayus, Phylicia McCoy, BS, Yat Fung Joshua Ng, Jenna Schindele, BS, David J. Reinkensmeyer and Susan Shaw in Neurorehabilitation and Neural Repair

sj-pdf-4-nnr-10.1177_15459683221146995 – Supplemental material for Optimized Home Rehabilitation Technology Reduces Upper Extremity Impairment Compared to a Conventional Home Exercise Program: A Randomized, Controlled, Single-Blind Trial in Subacute Stroke Click here for additional data file. Supplemental material, sj-pdf-4-nnr-10.1177_15459683221146995 for Optimized Home Rehabilitation Technology Reduces Upper Extremity Impairment Compared to a Conventional Home Exercise Program: A Randomized, Controlled, Single-Blind Trial in Subacute Stroke by Veronica A. Swanson, Christopher Johnson, Daniel K. Zondervan, Nicole Bayus, Phylicia McCoy, BS, Yat Fung Joshua Ng, Jenna Schindele, BS, David J. Reinkensmeyer and Susan Shaw in Neurorehabilitation and Neural Repair

sj-pdf-6-nnr-10.1177_15459683221146995 – Supplemental material for Optimized Home Rehabilitation Technology Reduces Upper Extremity Impairment Compared to a Conventional Home Exercise Program: A Randomized, Controlled, Single-Blind Trial in Subacute Stroke Click here for additional data file. Supplemental material, sj-pdf-6-nnr-10.1177_15459683221146995 for Optimized Home Rehabilitation Technology Reduces Upper Extremity Impairment Compared to a Conventional Home Exercise Program: A Randomized, Controlled, Single-Blind Trial in Subacute Stroke by Veronica A. Swanson, Christopher Johnson, Daniel K. Zondervan, Nicole Bayus, Phylicia McCoy, BS, Yat Fung Joshua Ng, Jenna Schindele, BS, David J. Reinkensmeyer and Susan Shaw in Neurorehabilitation and Neural Repair

sj-tif-1-nnr-10.1177_15459683221146995 – Supplemental material for Optimized Home Rehabilitation Technology Reduces Upper Extremity Impairment Compared to a Conventional Home Exercise Program: A Randomized, Controlled, Single-Blind Trial in Subacute Stroke Click here for additional data file. Supplemental material, sj-tif-1-nnr-10.1177_15459683221146995 for Optimized Home Rehabilitation Technology Reduces Upper Extremity Impairment Compared to a Conventional Home Exercise Program: A Randomized, Controlled, Single-Blind Trial in Subacute Stroke by Veronica A. Swanson, Christopher Johnson, Daniel K. Zondervan, Nicole Bayus, Phylicia McCoy, BS, Yat Fung Joshua Ng, Jenna Schindele, BS, David J. Reinkensmeyer and Susan Shaw in Neurorehabilitation and Neural Repair

sj-tiff-2-nnr-10.1177_15459683221146995 – Supplemental material for Optimized Home Rehabilitation Technology Reduces Upper Extremity Impairment Compared to a Conventional Home Exercise Program: A Randomized, Controlled, Single-Blind Trial in Subacute Stroke Click here for additional data file. Supplemental material, sj-tiff-2-nnr-10.1177_15459683221146995 for Optimized Home Rehabilitation Technology Reduces Upper Extremity Impairment Compared to a Conventional Home Exercise Program: A Randomized, Controlled, Single-Blind Trial in Subacute Stroke by Veronica A. Swanson, Christopher Johnson, Daniel K. Zondervan, Nicole Bayus, Phylicia McCoy, BS, Yat Fung Joshua Ng, Jenna Schindele, BS, David J. Reinkensmeyer and Susan Shaw in Neurorehabilitation and Neural Repair

sj-tiff-3-nnr-10.1177_15459683221146995 – Supplemental material for Optimized Home Rehabilitation Technology Reduces Upper Extremity Impairment Compared to a Conventional Home Exercise Program: A Randomized, Controlled, Single-Blind Trial in Subacute Stroke Click here for additional data file. Supplemental material, sj-tiff-3-nnr-10.1177_15459683221146995 for Optimized Home Rehabilitation Technology Reduces Upper Extremity Impairment Compared to a Conventional Home Exercise Program: A Randomized, Controlled, Single-Blind Trial in Subacute Stroke by Veronica A. Swanson, Christopher Johnson, Daniel K. Zondervan, Nicole Bayus, Phylicia McCoy, BS, Yat Fung Joshua Ng, Jenna Schindele, BS, David J. Reinkensmeyer and Susan Shaw in Neurorehabilitation and Neural Repair
