# Supplementary material for: Optimized Home Rehabilitation Technology Reduces Upper Extremity Impairment Compared to a Conventional Home Exercise Program: A Randomized, Controlled, Single-Blind Trial in Subacute Stroke
Source: Neurorehabil Neural Repair. 2023 Jan 12;37(1):53–65. doi: 10.1177/15459683221146995 (PMC9896541; doi:10.1177/15459683221146995)

RehabStudio Exercise Library

Current library of exercises that can be performed with the standard FitMi system.

Hand Exercises

H1: Wrist Flexion

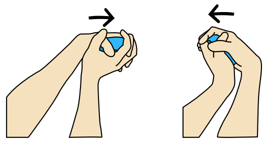

H2: Wrist Deviation

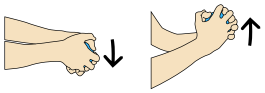

H3: Gripping

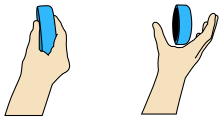

H4: Rotation

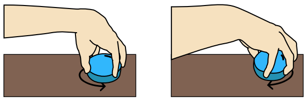

H5: Key Pinch Grip

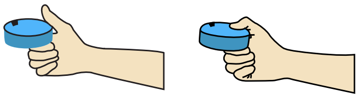

H6: Finger Tapping

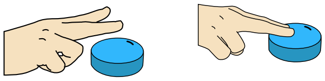

H7: Thumb Opposition

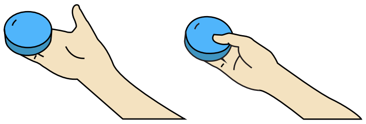

H8: Finger Twisting

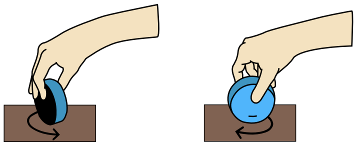

H9: Rolling

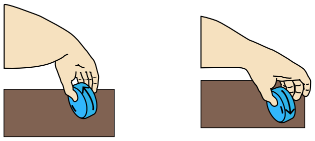

H10: Flipping

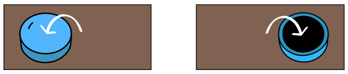

Arm Exercises

A1: Touches

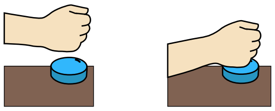

A2: Reach to Target #1

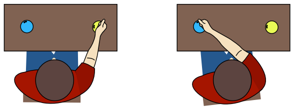

A3: Clapping

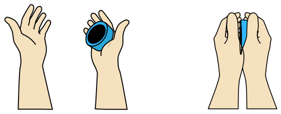

A4: Reach to Target #2

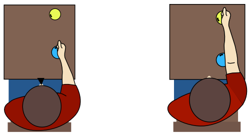

A5: Reach to Target #3

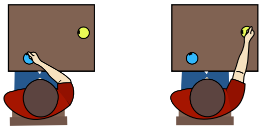

A6: Wrist Supination/ Pronation

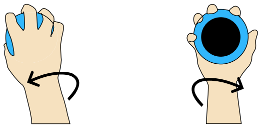

A7: Bicep Curls

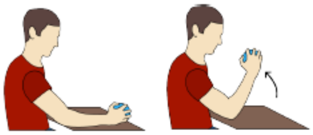

A8: Shoulder Flexion

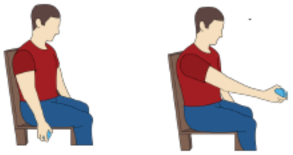

A9: Shoulder Abduction

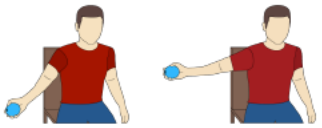

A10: Shoulder Rotation

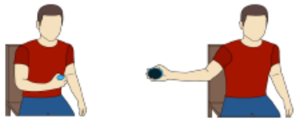

Trunk Exercises

T1: Weight Bearing - Center

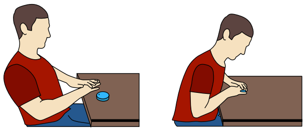

T2: Weight Bearing - Left Side

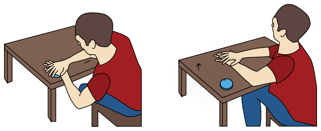

T3: Weight Bearing - Right Side

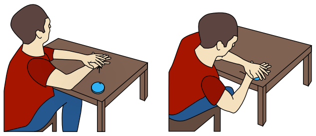

T4: Seated Twists

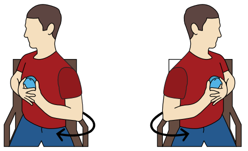

T5: Weight Bearing - Forward

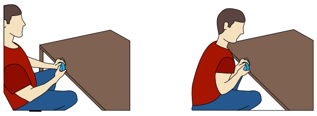

T6: Weight Bearing - Arms Out

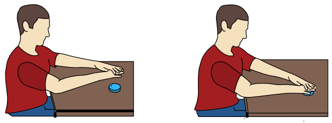

T7: Oblique Crunches

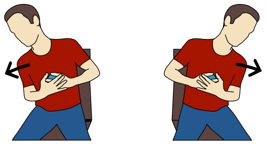

T8: Seated Crunches

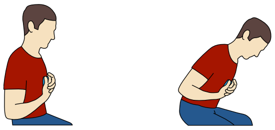

T9: Standing Weight Bearing Lean

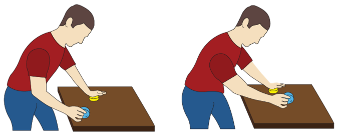

T10: Extended Twists

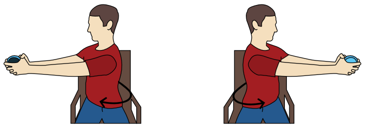

Leg Exercises

L1: Stomps

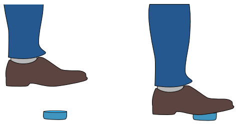

L2: Knee Squeeze

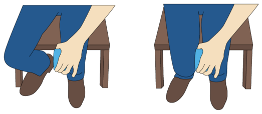

L3: Step Across

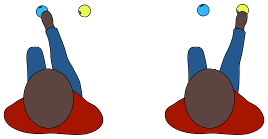

L4: Toe Taps

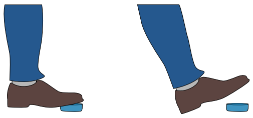

L5: Marching

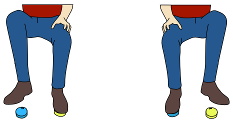

L6: Step Wide

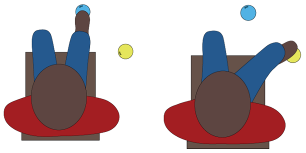

L7: Heel Taps

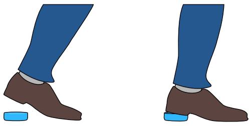

L8: Step Out

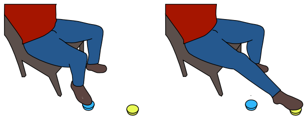

L9: Ankle Rotation

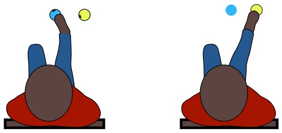

L10: Hip Flexion

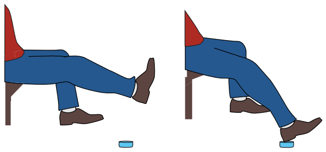

Supplement: sj-pdf-4-nnr-10.1177_15459683221146995 – Supplemental material for Optimized Home Rehabilitation Technology Reduces Upper Extremity Impairment Compared to a Conventional Home Exercise Program: A Randomized, Controlled, Single-Blind Trial in Subacute Stroke [file sj-pdf-4-nnr-10.1177_15459683221146995.pdf]
