# Supplementary material for: Optimized Home Rehabilitation Technology Reduces Upper Extremity Impairment Compared to a Conventional Home Exercise Program: A Randomized, Controlled, Single-Blind Trial in Subacute Stroke
Source: Neurorehabil Neural Repair. 2023 Jan 12;37(1):53–65. doi: 10.1177/15459683221146995 (PMC9896541; doi:10.1177/15459683221146995)
Supplement: sj-pdf-6-nnr-10.1177_15459683221146995 – Supplemental material for Optimized Home Rehabilitation Technology Reduces Upper Extremity Impairment Compared to a Conventional Home Exercise Program: A Randomized, Controlled, Single-Blind Trial in Subacute Stroke [file sj-pdf-6-nnr-10.1177_15459683221146995.pdf]

Study Protocol and Statistical Analysis Plan

for

RehabTouch: A Mixed-Reality Gym for Rehabilitating the Hands, Arms, Trunk, and Legs after Stroke

NCT03503617

Document Date: 08/31/2021

Trial Design

This study was a single-site, single-blind randomized controlled trial comparing home-based therapy with RehabTouch to conventional therapy for individuals in the subacute phase of stroke. RehabTouch is a commercial home rehabilitation technology (sold as FitMi® by Flint Rehab) designed to put into practice clinically recommended design features for at-home rehabilitation technology. The study was performed at Rancho Los Amigos National Rehabilitation Center in Downey, CA. Participants were invited for an initial assessment to confirm they met the inclusion criteria and to establish baseline measures. Participants provided informed written consent. Qualifying participants were randomly assigned to either the RehabTouch group or the conventional group. Participants in both groups were instructed to perform self-guided therapy for at least three hours/week for three consecutive weeks. All participants received weekly phone calls from a supervising therapist. After the three-week exercise period, participants returned for an end-of-therapy assessment and to return study materials. Participants returned one month later for a follow-up assessment. The trial was pre-registered on ClinicalTrials.gov (NCT03503617) and approved by the Rancho Research Institute, Inc. Institutional Review Board at Rancho Los Amigos National Rehabilitation Center (IRB #263).

Participants

Inclusion criteria were: experienced one or more strokes between 2 weeks and 4 months prior; baseline UEFM Score >5 and ≤55 out of 66; absence of moderate to severe pain defined as a score of 4 or lower on the 10 point visual-analog pain scale; ability to understand the instructions to operate RehabTouch; and aged 18 to 85 years old, as older age could be a confounding variable. Exclusion criteria were: concurrent severe medical problems that precluded the individual from participating in routine rehabilitation; visual deficits defined as a score >1 on question 3 of the NIH Stroke Scale (NIHSS); severe cognitive deficits or apraxia defined as a score >0 on questions 1a and 1c of the NIHSS; severe neglect defined as a score >1 on question 11 of the NIHSS; severe aphasia defined as a score >1 on question 9 of the NIHSS; and enrollment in other therapy studies. Recruitment aimed to balance the age, ethnicity, and gender of the study participants to be representative of Los Angeles County in California, USA. All participants provided informed consent.

Using an estimated effect size of 1.05 based on long-term follow-up data from a previous arm training study during subacute stroke,[1] power analysis established that 21 participants in each group would provide a 90% chance of detecting a significant difference between RehabTouch and conventional therapy at the 0.05 significance level (two-tailed t-test). To account for 20% dropout, the target sample size was n = 25 participants in each group.

Adaptive randomization was used to ensure matched levels of impairment between the RehabTouch and conventional therapy groups. Specifically, subjects were stratified by their UEFM Score into three levels (i.e. 5-22, 23-39, 40-55) and then randomized by alternating block allocation.[2]

Intervention

Participants randomized to the RehabTouch group were given a RehabTouch system with the custom 10” touchscreen tablet. They received 30 minutes of training on how to set up and use the RehabTouch system. They were instructed to spend most of their time performing upper extremity exercises, but access to the trunk and leg exercises in the RehabTouch software was not disabled.

Participants randomized to the conventional therapy group were given a booklet of paper exercises that were selected from the same library of 40 exercises available in the RehabTouch software.

For both groups, a supervising rehabilitation therapist selected the exercises for each participant based on their specific impairments. All participants received 30 minutes of training from the therapist on how to perform the selected exercises correctly.

After the 3-week exercise period, participants returned for an end-of-therapy assessment. At this assessment, participants returned the RehabTouch system or the sensorized booklet of exercises for data collection. Participants returned one month later for a follow-up assessment.

Outcomes

The primary outcome measure was the change in Upper Extremity Fugl-Meyer (UEFM) score[3] from baseline assessment to one-month follow-up. UEFM was assessed at baseline, end-of-therapy, and one-month follow-up. Secondary measures included the Box and Blocks Test,[4] the 10 Meter Walk Test,[5] the Modified Ashworth Spasticity (MAS) scale[6] for the elbow, wrist, and fingers, and the Visual Analog Pain (VAP) scale for the upper extremity, all of which were assessed at baseline, end-of-therapy, and one-month follow-up. Motor Activity Log (MAL) was measured at end-of-therapy and one-month follow-up to assess self-reported quantity and quality of movement.[7] The European Quality of Life five dimensions, three levels (EQ-5D-3L) and its companion Visual Analog Scale (EQ-VAS) were measured at end-of-therapy and at one-month follow-up to assess overall perceived health state,[8], [9] and the Intrinsic Motivation Inventory (IMI)[10] categories of Interest/Enjoyment, Value/Usefulness, and Effort/Importance were measured at end-of-therapy to assess participants’ perceived motivation. These measures are widely used in stroke rehabilitation research and have good sensitivity and reliability. All assessments were performed by a blinded, trained evaluator.

To assess adherence, the RehabTouch software recorded the date, time, and number of repetitions completed for each exercise, and the exercise booklet was placed in a sensorized folder that measured the times at which the participants opened the booklet.

Statistical Methods

Statistical analyses were performed using Matlab R2020 software. Change in UEFM score from baseline assessment to one-month follow-up was compared between the RehabTouch group and conventional therapy group using an unpaired two-tailed t-test. Three participants in the conventional therapy group did not perform their one-month follow-up assessment; one of these did perform the end-of-therapy assessment. For this one participant, the missing one-month follow-up data point was imputed for the primary outcome measure by adding the average change in UEFM score across all participants in the conventional therapy group from end-of-therapy to one-month follow-up to this participant's end-of-therapy UEFM score. All other analyses were performed using only data from participants who completed all three assessments.

MAS scores were grouped by flexion or extension items and summed to obtain lumped MAS extension and flexion values. We quantified items marked with a '+,' with an additional 0.5 points for calculations. EQ-5D-L3 was analyzed following.[8], [9] IMI categories were compared across groups using Wilcoxon rank sum tests. For the secondary outcomes with baseline assessments, differences between groups at baseline and at one-month follow-up were assessed using unpaired, two-tailed t-tests. Within group changes from baseline to end-of-therapy and baseline to one-month follow-up were compared using paired, two-tailed t-tests. For the secondary outcomes without baseline assessments, end-of-therapy scores were compared to one-month follow-up scores using paired, two-tailed t-tests.

To assess the ability of RehabTouch to motivate an appropriately high dose of home therapy, we performed a post-hoc exploratory analysis comparing the total number of repetitions that RehabTouch participants completed to a theoretical target dose of 2,700 repetitions. A dose of 2,700 repetitions of UE exercise corresponds to 300 repetitions/hour (5 reps/minute) over 9 hours of exercise, an intensity and duration sufficient to provoke a forelimb rehabilitative effect in a rodent model of stroke.[11]

Certain participants were missing data at one or more time points for the secondary outcomes MAS, VAP and IMI. If a participant's record for a given measure was missing data, that participant was omitted from any analysis for that measure. Thus, for MAS calculations (RehabTouch n=13 and conventional n=8), for VAP analysis (RehabTouch n=12 and conventional n=9), and for IMI analysis (RehabTouch n=13, and conventional n=11).

Interim Analysis

Due to the unexpected additional risks to participating in this study due to the COVID-19 pandemic, an unplanned interim futility/efficacy analysis of the primary outcome measure was conducted after recruitment was halted in March 2020. Group labels were removed, and the

analysis was reviewed by an independent investigator. For the futility analysis, a conditional power of 20% was selected. For the efficacy analysis, a P-value of 0.033 was selected using the Lan-DeMets alpha spending function for the Pocock boundary (n=27 out of a planned 50 at interim analysis).[12]

References Cited

[1] H. M. Feys *et al.*, "Effect of a therapeutic intervention for the hemiplegic upper limb in the acute phase after stroke: a single-blind, randomized, controlled multicenter trial," *Stroke*, vol. 29, no. 4, pp. 785–792, 1998.

[2] D. Machin, "Clinical trials: Design, conduct, and analysis. Curtis L. Meinert (1986) Monographs in epidemiology and biostatistics. Volume 8. Oxford University Press. New York and Oxford. Pages 469. £55 ISBN 5035682," *Hum. Psychopharmacol. Clin. Exp.*, vol. 3, no. 2, pp. 153–153, 1988.

[3] J. See *et al.*, "A standardized approach to the fugl-meyer assessment and its implications for clinical trials.," *Neurorehabil. Neural Repair*, vol. 27, no. 8, pp. 732–41, Oct. 2013.

[4] T. Platz, C. Pinkowski, F. van Wijck, I.-H. Kim, P. di Bella, and G. Johnson, "Reliability and validity of arm function assessment with standardized guidelines for the Fugl-Meyer Test, Action Research Arm Test and Box and Block Test: a multicentre study," *Clin. Rehabil.*, vol. 19, no. 4, pp. 404–411, Jun. 2005.

[5] F. M. Collen, D. T. Wade, and C. M. Bradshaw, "Mobility after stroke: reliability of measures of impairment and disability.," *Int. Disabil. Stud.*, vol. 12, no. 1, pp. 6–9.

[6] R. W. Bohannon and M. B. Smith, "Interrater reliability of a modified Ashworth scale of muscle spasticity," *Phys Ther*, pp. 206–207, 1987.

[7] J. H. van der Lee, H. Beckerman, D. L. Knol, H. C. W. de Vet, and L. M. Bouter, "Clinimetric properties of the Motor Activity Log for the assessment of arm use in hemiparetic patients," *Stroke*, vol. 35, no. 6, pp. 1404–1410, 2004.

[8] "Calculating the U.S. Population-based EQ-5D Index Score | AHRQ Archive." .

[9] J. W. Shaw, J. A. Johnson, and S. J. Coons, "US valuation of the EQ-5D health states: development and testing of the D1 valuation model," *Med. Care*, vol. 43, no. 3, pp. 203–220, Mar. 2005.

[10] *Intrinsic Motivation Inventory (IMI) – selfdeterminationtheory.org*. .

[11] R. L. Birkenmeier, E. M. Prager, and C. E. Lang, "Translating animal doses of task-specific training to people with chronic stroke in 1-hour therapy sessions: a proof-of-concept study.," *Neurorehabil. Neural Repair*, vol. 24, no. 7, pp. 620–35, Sep. 2010.

[12] D. L. DeMets and K. K. Lan, "Interim analysis: the alpha spending function approach," *Stat. Med.*, vol. 13, no. 13–14, pp. 1341-1352; discussion 1353-1356, Jul. 1994.
