# Supplementary figures and images for: Optimized Home Rehabilitation Technology Reduces Upper Extremity Impairment Compared to a Conventional Home Exercise Program: A Randomized, Controlled, Single-Blind Trial in Subacute Stroke
Source: Neurorehabil Neural Repair. 2023 Jan 12;37(1):53–65. doi: 10.1177/15459683221146995 (PMC9896541; doi:10.1177/15459683221146995)

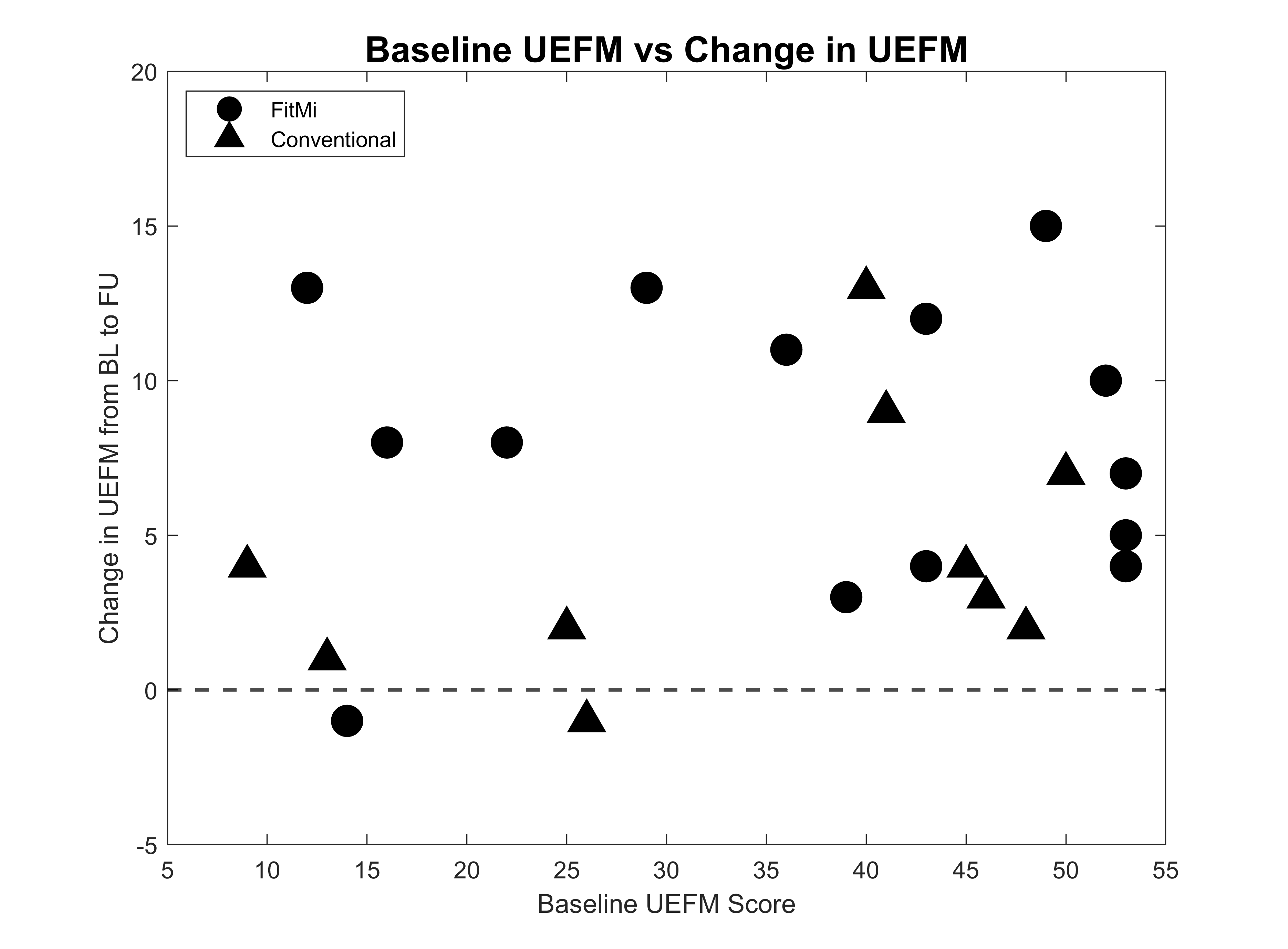

Supplement: sj-tif-1-nnr-10.1177_15459683221146995 – Supplemental material for Optimized Home Rehabilitation Technology Reduces Upper Extremity Impairment Compared to a Conventional Home Exercise Program: A Randomized, Controlled, Single-Blind Trial in Subacute Stroke [file sj-tif-1-nnr-10.1177_15459683221146995.tif]

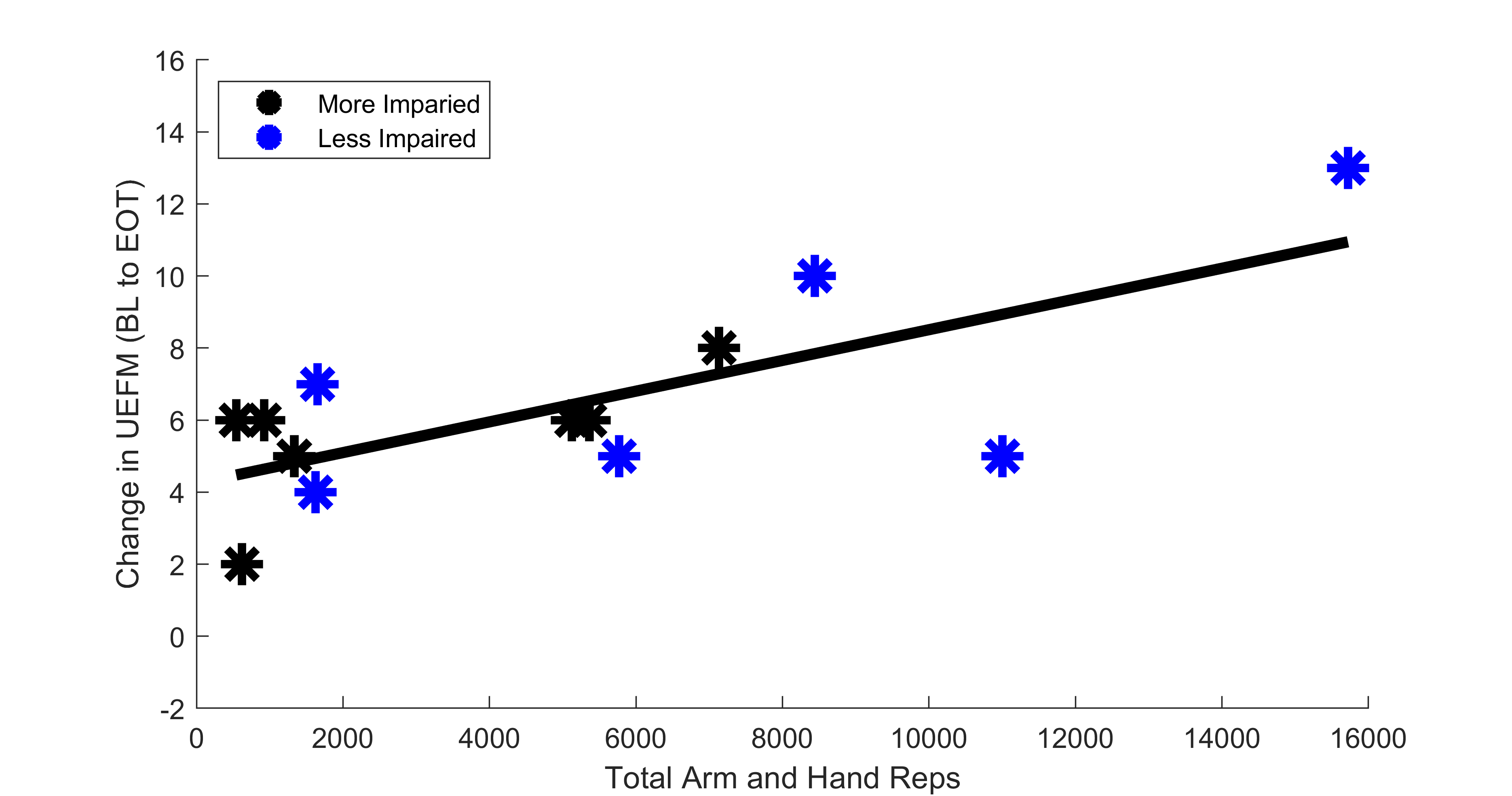

Supplement: sj-tiff-2-nnr-10.1177_15459683221146995 – Supplemental material for Optimized Home Rehabilitation Technology Reduces Upper Extremity Impairment Compared to a Conventional Home Exercise Program: A Randomized, Controlled, Single-Blind Trial in Subacute Stroke [file sj-tiff-2-nnr-10.1177_15459683221146995.tiff]

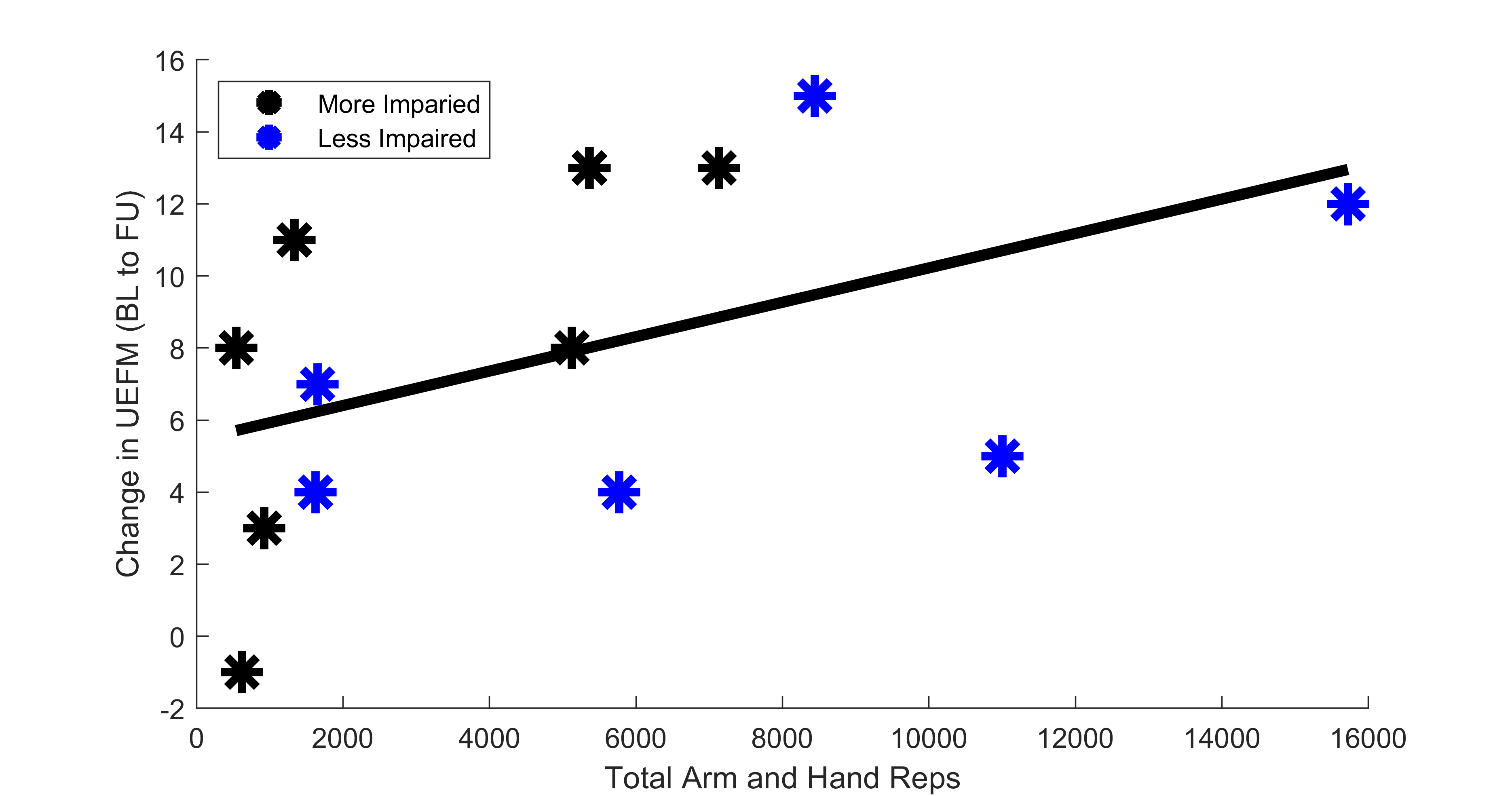

Supplement: sj-tiff-3-nnr-10.1177_15459683221146995 – Supplemental material for Optimized Home Rehabilitation Technology Reduces Upper Extremity Impairment Compared to a Conventional Home Exercise Program: A Randomized, Controlled, Single-Blind Trial in Subacute Stroke [file sj-tiff-3-nnr-10.1177_15459683221146995.tiff]
